# Tributyltin Alters Seric Bile Acid Pool Composition in Male Rats

**DOI:** 10.3390/toxics13060440

**Published:** 2025-05-26

**Authors:** Wenhuan Yao, Jinjiao Luan, Hui Li, Dong Cheng, Shibo Lv, Jiliang Si

**Affiliations:** 1 Institute of Preventive Medicine, School of Public Health, Cheeloo College of Medicine, Shandong University, Jinan 250012, China; fengyaoyao96@163.com (W.Y.); luanjinjiao@163.com (J.L.); lwlh163@163.com (H.L.); sdchd@aliyun.com (D.C.); 18615281861@wo.cn (S.L.); 2 Department of Toxicology, Shandong Center for Disease Control and Prevention, Jinan 250014, China

**Keywords:** tributyltin, bile acids, liver, UHPLC-MS/MS

## Abstract

Tributyltin (TBT), a recognized endocrine disruptor, is associated with metabolic diseases, including obesity, type 2 diabetes, non-alcoholic steatohepatitis, and osteoporosis. Bile acids (BAs) play pivotal roles in lipid digestion and absorption. However, there are no studies to illustrate the effects of TBT on BA pool composition in circulation. Here, rats were treated with TBT (50 μg/kg) or a vehicle control once every three days for sixty days to analyze serum BA levels using ultra-high-performance liquid chromatography–tandem mass spectrometry (UHPLC-MS/MS). The liver tissue sections and lipid levels of rats were examined using conventional methods. TBT induced sporadic cholestasis in the livers of rats and significantly reduced the levels of five BAs, including four conjugated BAs [acidtaurocholic acid (TCA), taurodeoxycholic acid (TDCA), taurochenodeoxycholic acid (TCDCA), and tauro-β-muricholic acid (Tβ-MCA)] and one unconjugated bile acid [dehydrolithocholic acid (DLCA)], while the serum levels of triglyceride, cholesterol, and bilirubin were unaltered by TBT treatment. These results indicate that TBT exposure affected the BA pool composition in circulation, especially the taurine-conjugated BAs.

## 1. Introduction

Tributyltin (TBT) was widely utilized in wood preservation, industrial water disinfection, antifouling coatings for ships, and slime control in paper mills [1]. Once released from antifouling coatings, TBT rapidly binds to organic matter, such as bacteria and algae, or adsorbs on suspended particles in aquatic systems [2]. These particles are subsequently ingested by filter-feeding zooplankton and herbivorous invertebrates, facilitating bioaccumulation and biomagnification across trophic levels, ultimately affecting fish, seabirds, and mammals [3,4]. Although the International Maritime Organization (IMO) globally banned TBT, its environmental persistence and lipophilic properties continue to pose significant ecological and toxicological risks [5]. A recent systematic review and meta-analysis revealed that the overall average estimate for TBT in seafood was 182.33 μg·kg^−1^, based on surveys conducted in Europe, America, and Asia [6]. Furthermore, elevated levels of TBTs have been detected in human liver tissue (59–96 μg/kg wet weight) [7]. Transcriptomic analysis revealed that TBT could interfere with lipid metabolism both in mammals and aquatic gastropods [5,8]. Many mammalian experiments have provided evidence that TBT can induce metabolic syndromes such as obesity and dyslipidemia [9,10,11,12].

Bile acids (BAs), steroidal molecules synthesized in the liver, are critical regulators of lipid digestion and absorption [13]. Beyond their classical roles in enterohepatic circulation, BAs function as signaling molecules, modulating glucose and lipid metabolisms through nuclear receptors such as farnesoid X receptor (FXR) and G-protein-coupled receptor (TGR5) [14,15]. However, there are no studies to illustrate the effects of TBT on BA pool composition in circulation. Here, we used UHPLC-MS/MS to characterize serum BA profiles and to analyze how their composition is altered by TBT.

## 2. Materials and Methods

### 2.1. Reagents

Tributyltin chloride (TBTCl, ≥96% purity) was purchased from TCI Development Co., Ltd. (Shanghai, China). Methanol, acetonitrile, isopropanol, and acetic acid were LC/MS grade and were obtained from Merck Life Science (Shanghai, China) Co., Ltd. Standard compounds were sourced from Cayman Chemical Company (Ann Arbor, MI, USA). The hepatobiliary pigment stain kit was purchased from Beijing Solarbio Science & Technology Co., Ltd. (Beijing, China). All other chemicals used in this study were of analytical grade and were obtained from commercial sources.

### 2.2. Animals and Treatment

Twenty-one-day-old male Sprague-Dawley (SD) rats (body weight 55–65 g) were purchased from Jinan Pengyue Laboratory Animal Breeding Co., Ltd. [License No. SCXK (Lu) 20190003]. The animals were housed in an air-conditioned room with controlled temperature (23 °C ± 2 °C) and humidity (55% ± 15%). They were kept in plastic cages lined with sawdust under a 12 h light–dark cycle, with food and water provided ad libitum.

All experimental procedures were conducted in accordance with the National Institutes of Health (NIH) Guide for the Care and Use of Laboratory Animals and adhered to the principles outlined in the “Use of Animals in Toxicology”. The study protocol was approved and supervised by the Ethics Committee of the School of Public Health, Shandong University.

After a three-day acclimatization period, rats were randomly assigned into two groups (*n* = 6 per group) based on body weight to ensure similar average weights across groups. From postnatal day (PND) 24 to PND 84, rats were administered corn oil or 50 μg/kg TBT via gavage every three days. Here, 50 μg/kg TBT is approximately double the established no-observable-adverse-effect level of 25 μg/(kg·day) [16]. However, the administration frequency was every three days instead of once daily.

### 2.3. Preparation of Serum and Tissue Isolation

On PND 85, one day after the final gavage, the rats were anesthetized with urethane (~1.2 g/kg) via intraperitoneal injection. Once the surgical level of anesthesia was achieved, blood was collected from the ventral aorta. The serum was separated and stored at –80 °C for subsequent analysis. The left lobe of the liver from each animal was excised, fixed in 4% (*v*/*v*) paraformaldehyde, and stored at 4 °C overnight. The remaining liver tissue was sectioned, flash-frozen in liquid nitrogen, and stored at –80 °C for other analysis.

### 2.4. Histopathological Analysis

Formalin-fixed specimens were routinely processed. After being fixed for 12 h, they were embedded in paraffin and sectioned into 4 μm thick slices. Hematoxylin and eosin (H&E) staining and hepatobiliary pigment staining were performed using standard procedures for morphological observations.

### 2.5. Serum Biochemistry

Serum triglycerides (TGs), cholesterol (CHOL), and bilirubin were measured using an autoanalyzer (Beckman Coulter AU480, Beckman Coulter, Inc., Brea, CA, USA).

### 2.6. Analysis of Serum BAs

BA analyses were performed according to a previously reported method [17], with minor modifications. Briefly, 50 μL serum samples were thawed on ice and spiked with internal standards. The samples were vortexed for 10 min and then incubated at −20 °C for another 10 min. Following centrifugation at 12,000 rpm for 10 min at 4 °C, the supernatants were transferred to clean tubes and dried using a Savant SpeedVac concentrator (Thermo Fisher Scientific, Waltham, MA, USA). The dried residues were reconstituted in 100 μL of methanol/water (50:50, *v*/*v*) and prepared for LC-MS/MS analysis.

Concentrations of BAs in rat serum were quantified using ultra-high-performance liquid chromatography (UHPLC, Shim-pack UFLC SHIMADZU CBM30A system) with tandem mass spectrometry (Applied Biosystems 6500 QTRAP, Thermo Fisher Scientific, Waltham, MA, USA). For chromatography, chromatographic separation was achieved using an Acquity UPLC HSS T3 C18 column (1.8 µm, 2.1 × 100 mm). The mobile phase consisted of 5 mM ammonium acetate with 0.01% formic acid in water (phase A) and 0.01% formic acid in acetonitrile (phase B). The column temperature was maintained at 40 °C, and the gradient elution program began with 5% phase B held for 0.5 min, increased linearly to 40% in 0.5 min, then to 50% over 4.5 min, to 80% over 7.5 min, and finally to 95% at 10 min, followed by a 12 min re-equilibration to the starting conditions. The flow rate was set at 0.35 mL/min. Mass spectrometric analysis was performed using tandem mass spectrometry (Applied Biosystems 6500 QTRAP) equipped with an electrospray ionization (ESI) source operating in negative ion mode and utilizing multiple reaction monitoring (MRM). The ion source temperature was set to 550 °C. EPI (echo planar imaging) was performed from *m*/*z* 50 to 640 at a speed of 1000 da/s.

Data from mass spectrometry were analyzed using the Metware Database (https://cloud.metware.cn). The relative abundance of BAs was calculated as the ratio of the peak area of each respective BA to its corresponding internal standard. BA standard solution concentrations were measured using LC-MS/MS, and linear regression equations were generated by plotting the standard content (*x*-axis) against the peak area ratio between the standard and its internal standard (*y*-axis). This approach enabled the quantitative determination of BA concentrations in each sample.

### 2.7. Statistical Analysis

Differential analysis of BAs between the control and TBT groups was performed using principal component analysis (PCA), hierarchical cluster analysis, and partial least squares-discriminant analysis (PLS-DA). The normality of the data was evaluated with the Shapiro–Wilk test, and homogeneity of variances was tested using Levene’s test. If the distribution was normal, the *t*-test was used to determine the difference in means between the two groups. If not, the Mann–Whitney U test was applied. Statistical analyses were performed using Rstudio software (version 4.2.3; Posit Inc., Boston, MA, USA). All tests were two-sided, with statistical significance set at a *p*-value less than 0.05.

## 3. Results

### 3.1. Effects of TBT Exposure on Systemic Toxicity and Lipid Metabolism in Rats

**Figure 1:**
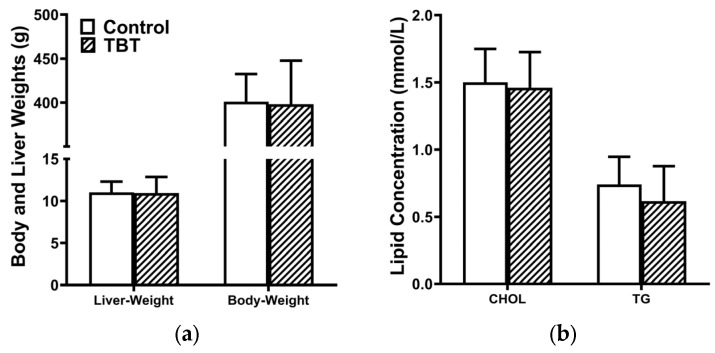
TBT did not alter the development and serum lipid levels of rats. (**a**) Body and liver weights and (**b**) serum levels of triglycerides (TGs) and cholesterol (CHOL) in rats were not affected by TBT on PND 85. Data are presented as means ± SEMs (*n* = 6 per group).

As illustrated in Figure 1a, no significant differences in body weight or liver weight were observed between the control and TBT groups. In addition, the serum levels of TGs and CHOL were comparable between the two groups (Figure 1b).

### 3.2. Effects of TBT Exposure on the Liver in Rats

Although the serum bilirubin levels in rats were elevated in the TBT group (P25 = 0, P50 = 0.04, and P75 = 0.30 μmol/L for the control; P25 = 0.15, P50 = 0.22, and P75 = 0.48 μmol/L for the TBT group), the difference was not statistically significant (*p* = 0.072).

**Figure 2:**
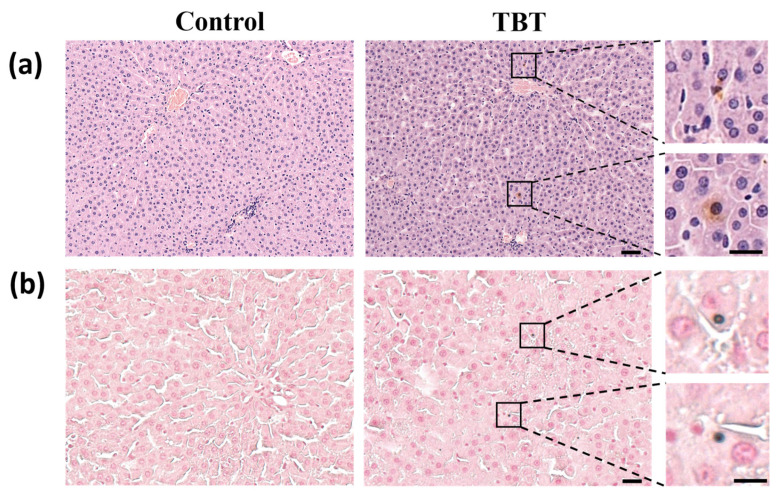
TBT caused mild cholestasis in rat livers. (**a**) Representative images of rat liver sections stained with hematoxylin and eosin (H&E). Sporadic brown staining spots show the color of BAs in the livers of the rats. Scale bars: 50 μm, *n* = 6. On the right is a magnification of local cholestasis in the liver section of the TBT group. Scale bars: 20 μm. The upper inset highlights BA deposition at the edge of the hepatocyte, while the lower inset shows BA accumulation within hepatocytes. (**b**) Representative images of slices stained with modified Fouchet’s method for hepatobiliary bilirubin. Scale bars: 25 μm, *n* = 6. On the right is a magnification of the local hepatocyte bilirubin staining in the liver section of the TBT group, showing bilirubin within the hepatocyte. Scale bars: 10 μm.

Histopathological analysis of liver sections of H&E staining (Figure 2a) showed no detectable hepatocyte damage. However, sporadic brown staining spots were observed within hepatocytes on liver sections from the TBT group, which were absent in the control group. The following Fouchet’s stain demonstrates that TBT treatment increased the frequency of bile acids present in hepatocytes within the rat liver (Figure 2b). These data indicate that rats in the TBT group exhibited mild cholestasis.

### 3.3. Effects of TBT on Serum BAs

The concentrations of 50 BAs in rat serum were measured using UHPLC-MS/MS. In the analysis of all samples, 15 BAs had concentrations below the detection limit (LOD) of the method, while 3 others were below the LOD in some of the samples (Table S1). Consequently, these BAs were excluded from further analysis. The total ion chromatogram (TIC) and the multimodal plots generated through multiple reaction monitoring (MRM) are shown in Figures S1 and S2, respectively.

**Figure 3:**
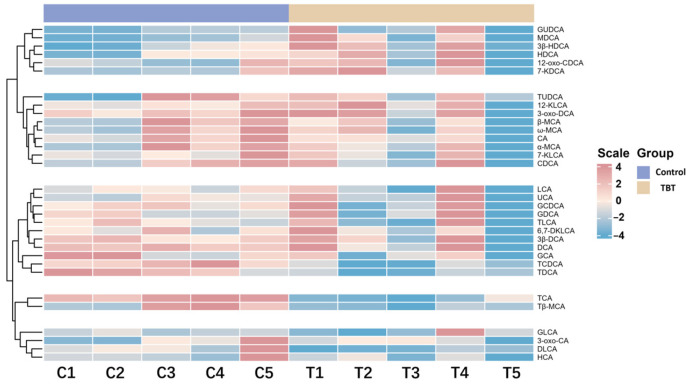
TBT perturbed the BA profile in rat serum. This heatmap shows the relative concentrations of BAs in the control and TBT groups (*n* = 5 *). Each row represents a specific BA, and each column represents an individual sample. Red indicates higher BA levels, while blue indicates lower levels. A clustering analysis reveals distinct groupings between the control and TBT groups, highlighting significant alterations in the BA profile following TBT exposure. * One serum sample from the control group was found to be slightly hemolytic during the quality inspection; thus, this sample and the corresponding one from the treatment group were rejected. Consequently, the sample size for the experiment was *n* = 5 per group.

The clustering analysis revealed distinct groupings between the control and TBT groups of rats (Figure 3), demonstrating significant alterations in BA profiles.

### 3.4. The Results of Differences in BA Analysis Between Groups

**Figure 4:**
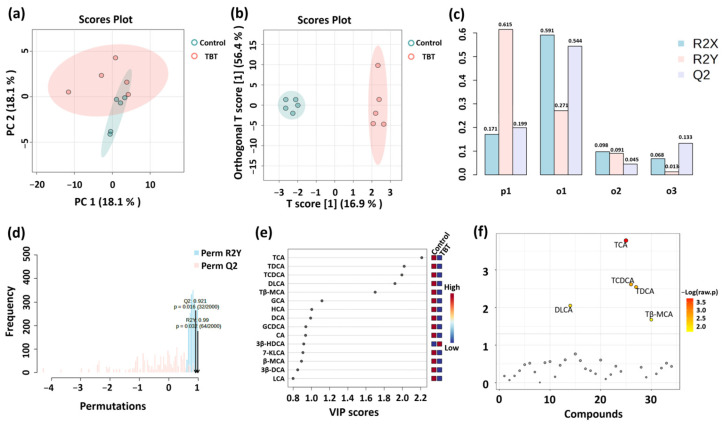
Analysis of serum BA differences between groups (*n* = 5 per group). (**a**) PCA was initially employed for exploratory analysis to assess group clustering and separation trends. (**b**) A supervised OPLS-DA model was applied to identify differential metabolites between groups, which were assessed using the values of R^2^X, R^2^Y, and Q^2^ (**c**). (**d**) Permutation testing (*n* = 2000) was used to confirm the validity of the OPLS-DA model. R^2^X and R^2^Y represent the proportion of variance explained by the model for the independent variable (X) and dependent variable (Y), respectively; values closer to 1 indicate a better model fit. Q^2^ is a key indicator of predictive performance, with Q^2^ > 0.5 suggesting a reliable model. VIP (**e**) and *t*-tests (**f**) were used to demonstrate BA levels in the serum. The variable importance in projection (VIP) score quantifies the contribution of each variable to the OPLS-DA classification, with VIP > 1.0 indicating greater importance. −Log (raw *p*) represents the negative logarithm of the *p*-value from the *t*-test, reflecting the statistical significance of BA level differences between groups; smaller *p*-values correspond to larger −Log (raw *p*) values.

To further explore the impact of TBT on BA composition, multiple statistical analyses were conducted to identify metabolic differences between groups. PCA was initially employed for exploratory analysis to assess group clustering and separation trends, visualizing the similarities or differences in BA levels between groups. Two principal components were retained, with PC1 and PC2 explaining 62.4% and 17.4% of the total variance, respectively. As shown in Figure 4a, samples were separated into two groups based on TBT treatment, demonstrating a significant impact of TBT on BA levels.

Subsequently, a supervised OPLS-DA model was applied to identify differential BAs between groups. This model offers superior classification efficiency compared to PCA by filtering system noise and extracting relevant variable information. As illustrated in Figure 4b, samples from the control and TBT groups were distinctly clustered, indicating significant differences in BA levels between groups. The values of R^2^X, R^2^Y, and Q^2^ in the OPLS-DA model were 0.171, 0.615, and 0.199, respectively, indicating the excellent capability and predictability of the model (Figure 4c). Additionally, permutation testing (*n* = 2000) confirmed that the OPLS-DA model was not overfitted and exhibited excellent predictive performance (Q^2^ = 0.921, R^2^Y = 0.99; Figure 4d). VIP was used to analyze differential BAs, which contribute to the variation between groups (Figure 4e).

**Figure 5:**
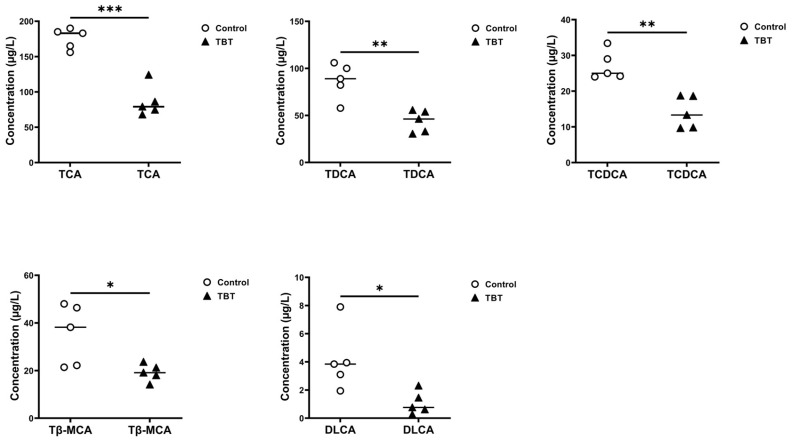
TBT exposure reduced BA concentrations in rat serum. Dot plots showing the BAs with different concentrations in the sera of rats from the control and TBT groups, including TCA, TDCA, TCDCA, Tβ-MCA, and DLCA. Each point represents an individual sample, with horizontal lines indicating group mean values (two-tailed *t*-tests, *n* = 5 per group). * *p* < 0.05, ** *p* < 0.01, and *** *p* < 0.001 compared with the control.

Independent sample *t*-tests were performed to identify significant differences in BAs between the control and TBT groups (Figure 4f), and five BAs with significant differences were finally screened. As further shown in Figure 5, taurocholic acid (TCA) significantly reduced the levels by 50.88% (*p* = 0.00013), taurodeoxycholic acid (TDCA) by 50.24% (*p* = 0.00363), taurochenodeoxycholic acid (TCDCA, *p* = 0.00131) by 52.24% (*p* = 0.00131), tauro-β-muricholic acid (Tβ-MCA) by 46.00% (*p* = 0.04678), and dehydrolithocholic acid (DLCA) by 58.51% (*p* = 0.03476) in the TBT group.

## 4. Discussion

In this study, TBT did not significantly impact serum TG and CHOL levels in rats. This finding contrasts with our previous study, where male ICR mice were administered 50 μg/kg TBT once every three days via intraperitoneal injection (IP) [11]. This discrepancy may be attributed to differences in susceptibility due to both interspecies variations and exposure routes. Rats and mice possess distinct body surface area conversion factors (BSA-CFs), and IP injection circumvents the gastrointestinal tract, leading to a different pharmacokinetic profile compared to oral gavage. Sex also plays a critical role in the effects of TBT. An experimental study using adult male Wistar rats exposed to 100 ng/(kg·d) TBT via oral gavage for 15 days demonstrated a significant increase in serum LDL levels compared to controls [18], while another study involving adult female Wistar rats administered with a higher dose of TBT [0.1 mg/(kg·d)] via oral gavage for 15 days showed no significant changes in serum lipid levels [19]. These suggest that gender, the dose of treatment, exposure route, and strain of the animals might play critical roles in the effects of TBT on serum lipid levels.

Mild cholestasis was observed in the TBT-treated rats in the present study. Cholestasis is a pathological condition characterized by the obstruction of bile production or excretion, resulting in the abnormal accumulation of bile components within the liver. Under normal physiological conditions, primary BAs, cholic acid (CA), and chenodeoxycholic acid (CDCA) are synthesized from cholesterol by the action of cholesterol hydroxylase enzymes in the liver [20]. In rodents, CDCA is rapidly converted to the more hydrophilic MCAs [21]. Then, they are amidated by BA-CoA synthetase (BACS) and BA-CoA:amino acid N-acyltransferase (BAAT) with taurine to form TCA or TCDCA in rodents. BAs are transported from hepatocytes into the bile ducts via transporters such as the bile salt export pump (BSEP) and multidrug resistance-associated protein 2 (MRP2), ensuring the efficient flow of bile [22,23]. Factors hindering bile production or transport can induce cholestasis. The results of histopathological observation suggested that TBT treatment might affect BAs in rats.

Consequently, TBT reduced the abundance of TCA, TCDCA, and Tβ-MCA in the sera of rats. Conjugation significantly enhances the water solubility of BAs [22]. Inversely, decreased hydrophilicity of bile due to reduced levels of conjugated BAs directly limits substrate availability for the BSEP, leading to impaired bile secretion and the onset of cholestasis [24]. TBT treatment did not affect the levels of CA and CDCA but did reduce the levels of TCA and TCDCA in the present study, suggesting that TBT might impair the process of conjugation. The hampered ability of BACS or BAAT, or both, by TBT might be a reasonable explanation for the reduction in taurine-conjugated primary BAs.

Gut microbiota actively participates in the biotransformation of BAs, thereby modulating BA pool composition and enterohepatic circulation [20]. Once in the intestine, BAs are bio-transformed into secondary BAs through a series of reactions, such as deconjugation, 7α-dehydroxylation, 6α-hydroxylation, and epimerization [21]. Studies showed that TBT exposure not only induced gut microbiome dysbiosis [12,25,26] but also decreased the content of secondary Bas, including 7-ketoLCA, T-alpha-MCA, 12-ketoLCA, and alpha-MCA, in the feces of mice [26]. Nevertheless, we observed decreased levels of TDCA and DLCA in the sera of rats, whereas our previous research demonstrated that exposure to 50 μg·kg^−1^ TBT significantly reduced the abundance of key gut microbiota genera [25], such as Clostridium, Lactobacillaceae, and Bifidobacteriaceae, which are responsible for deconjugating taurine- and glycine-conjugated BAs and converting primary BAs into secondary BAs [22]. These data suggest that the reduced levels of taurine-conjugated BAs in TBT-treated rats are more likely due to the reduced activity of BACS and/or BAAT rather than TBT-induced dysbiosis of the gut microbiota. DLCA is essential for anti-inflammatory effects [14], illustrating that TBT may influence the immunity of rats by lowering DLCA levels.

A study indicated that taurine deficiency was a driver of aging, in which taurine levels in circulation declined with age in mice, monkeys, and humans; however, taurine supplementation reversed this decline, enhancing the health span and life span in mice and worms, as well as the health span in monkeys [27]. Lots of studies showed that TBT was associated with adverse health effects of aging, such as immunosenescence, osteoporosis, and metabolic syndrome [28,29,30,31]. Based on these data, we speculate that TBT exposure may accelerate aging processes in rats, resulting in a secondary deficiency of taurine. This taurine deficiency may, in turn, contribute to the observed decrease in serum taurine-conjugated bile acids. Further studies measuring serum taurine levels and aging-related biomarkers would be valuable to clarify this potential mechanistic link.

It is worth noting that BAs are not only critical for lipid digestion and absorption but also play a pivotal role in thyroid hormone (TH) metabolism via interaction with the TGR5 receptor [32]. Numerous studies have reported that TBT exposure disrupts TH homeostasis in both vertebrates [33,34,35,36] and invertebrates [37,38]. Type 2 iodothyronine deiodinase (D2), a key enzyme for TH activation, catalyzes the conversion of inactive thyroxine (T4) to active tri-iodothyronine (T3) [39]. D2 also serves as a critical determinant of TH receptor saturation in cells expressing this enzyme [32]. Major BA species such as CA, TCA, DCA, and CDCA could increase cAMP levels in a dose-dependent fashion, which parallels the upregulation of D2 activity via the TGR5 in rodent brown adipocytes and human skeletal myocytes [32]. Among these, TCA is particularly effective at equimolar concentrations [32]. While high concentrations of TCA stimulate D2 activity, levels below physiological concentrations, such as those observed in TBT-treated rats, might signify reduced D2 activity. In our study, the significant reduction in TCA levels in TBT-treated rats might have decreased D2 activity, impairing the activation of THs. Given the essential role of D2 in TH activation, this finding provides a plausible mechanism for the observed disruption of TH homeostasis induced by TBT exposure.

Notably, endogenous BAs share a common steroid nucleus but differ primarily in their hydroxylation patterns at different positions of the carbon structure, as well as in amidation at the carboxyl terminus [40]. Ligand-binding studies and mutagenesis scanning have revealed that TCA and TDCA interact with distinct residues of the TGR5 receptor [40], suggesting that the effects of TBT on TGR5 via BA signaling pathways are complex and multifaceted. Moreover, TBT exposure affected the BA pool composition in circulation, especially the taurine-conjugated BAs in this study. One recent study showed that conjugated BA levels decreased in cerebrospinal fluid from Alzheimer’s Disease patients with poor outcomes [41], suggesting that the role of BAs goes beyond digestion.

## 5. Conclusions

This study demonstrates that TBT significantly reduced the levels of five BAs, including TCA, TDCA, TCDCA, T β-MCA, and DLCA, in the sera of rats with mild cholestasis in their livers. These results indicate that TBT exposure affected the BA pool composition in circulation, especially the taurine-conjugated BAs, suggesting that TBT may specifically disrupt conjugation processes or transport mechanisms. Further studies are warranted to explore the underlying mechanisms and to evaluate the relevance of these findings in human models.

## Data Availability

The datasets used and analyzed during the current study are available from the corresponding author on reasonable request. All data generated or analyzed during this study are included in the published article.

## Supplementary Materials

The following supporting information can be downloaded at https://www.mdpi.com/article/10.3390/toxics13060440/s1: Figure S1: Multiple reaction monitoring (MRM) chromatogram of detected metabolites; Figure S2: Total ion current (TIC) chromatogram of quality control (QC) samples; Table S1: Serum bile acid levels in rats (μg/L) and the values of FC, P, and Log_2_FC.
