# Supplementary material for: Tributyltin Alters Seric Bile Acid Pool Composition in Male Rats
Source: Toxics. 2025 May 26;13(6):440. doi: 10.3390/toxics13060440 (PMC12196776; doi:10.3390/toxics13060440)
Supplement: Supplementary file 1 [file toxics-13-00440-s001.zip › Supplementary Materials.pdf]

Tributyltin Alters Seric Bile Acid Pool Composition in Male Rats

Table S1. Serum bile acid levels in rats (μg/L) and the values of P, FC, and Log_2FC.

Index	Compounds	C1	C2	C3	C4	C5	H1	H2	H3	H4	H5	P	FC	Log_2FC
12-KLCA	12-ketolithocholic acid	10.5	9.34	12.6	11.2	17.2	17.1	22.2	9.64	18.9	0.862	0.714343	1.1292	0.1753
12-oxo-CDCA	12-Oxochenodeoxycholic acid	13.7	10.8	5.94	5.16	29	25.6	28.4	3.74	35.6	0.6	0.499541	1.4542	0.5402
3-oxo-CA	3-Oxocholic acid	3.02	2.62	14	11.8	27.2	9.38	13.7	14.4	10.4	0.22	0.696207	0.8203	-0.2858
3-oxo-DCA	3-oxodeoxycholic acid	14.9	11.4	16.4	14.2	21.2	21.2	20.2	8.2	20	2.34	0.778568	0.9211	-0.1186
3β-DCA	3β-deoxycholic acid	44.6	49.2	34.2	32.6	42	55.6	39.6	12.4	60	4.64	0.625199	0.8501	-0.2343
3β-HDCA	β-Hyodeoxycholic Acid	10.7	13.9	42.8	58.2	65	115	90.8	30.6	109	14.7	0.198316	1.8893	0.9179
3β-UDCA	3β-Ursodeoxycholic Acid	7	6.98	N/A	N/A	N/A	N/A	N/A	N/A	N/A	N/A	N/A	N/A	N/A
6,7-DKLCA	6,7-diketolithocholic acid	8.96	7.2	13.4	4.82	9.5	15.7	8.06	4.64	10	1.61	0.789973	0.9118	-0.1332
7-KDCA	7-Ketodeoxycholic acid	39	35.6	46.4	41.8	130	159	177	57.4	139	1.71	0.248109	1.8241	0.8672
7-KLCA	7-ketolithocholic acid	11.6	9.5	13.4	10.9	26.2	18.9	11.6	2.9	21.6	0.492	0.552484	0.775	-0.3677
CA	Cholic acid	662	548	1370	958	1510	868	872	608	928	40.8	0.206219	0.6571	-0.6058
CDCA-3Gln	Chenodeoxycholic acid-3-β-D-glucuronide	N/A	N/A	2.22	2.22	10.6	4.92	3.4	0.706	5.92	N/A	0.703752	0.7453	-0.4241
CDCA	Chenodeoxycholic acid	418	398	992	1230	1370	1290	470	266	1460	12.3	0.61953	0.7936	-0.3335
DCA	Deoxycholic acid	416	388	460	378	380	528	268	183	530	45.4	0.387553	0.7687	-0.3795
DLCA	Dehydrolithocholic acid	3.1	3.94	3.84	1.94	7.9	0.264	0.61	0.762	2.3	1.45	0.034757	0.2599	-1.944
GCA	Glycocholic acid	83.2	84.8	34	31.4	54.8	61.4	9.72	38.2	58.4	6.74	0.201313	0.6053	-0.7243
GCDCA	Glycochenodeoxycholic acid	4.54	5.88	6.38	3.96	5.36	8.66	0.962	3	8.44	0.526	0.643914	0.8265	-0.2749
GDCA	Glycodeoxycholic acid	36.6	39.2	20.4	19.7	24.4	54.6	5.36	22.6	57.2	2.48	0.976317	1.0138	0.0198
GLCA	Glycolithocholic acid	0.518	0.664	0.412	0.5	0.556	0.27	0.128	0.312	1.41	0.568	0.975454	1.0143	0.0205

GUDCA	Glycoursodeoxycholic acid	4.14	3.8	12.1	12.5	12.8	44.8	7.22	11.7	40.8	1.46	0.252731	2.3375	1.225
HCA	hyocholic acid	10	9.92	8.58	5.14	27.6	9.18	12.9	2.7	11.4	0.864	0.330428	0.6049	-0.7252
HDCA	Hyodeoxycholic acid	272	246	1580	1550	1660	1890	2620	672	3000	79.6	0.394678	1.5564	0.6382
ILCA	isolithocholic acid	4.48	4.6	4.16	4.3	5.1	6.22	4.26	3.82	8.06	N/A	0.357428	1.2345	0.3039
LCA	Lithocholic acid	10.1	13.3	12.5	9.18	15	17.5	8.8	2.62	23.2	2.56	0.810174	0.9101	-0.1359
LCA-3S	lithocholic acid-3-sulfate	0.32	0.406	N/A	N/A	N/A	0.408	0.214	N/A	0.308	N/A	N/A	0.854	-0.2277
MDCA	murideoxycholic acid	6.08	7.38	20.4	24.4	42.4	119	71.2	11	78	2.4	0.177716	2.7975	1.4841
TCA	Taurocholic acid	165	156	183	190	185	79	74.6	67.8	86	124	0.000138	0.4908	-1.0268
TCDCA	Taurochenodeoxycholic acid	24.2	25	29	33.4	24	18.6	9.62	9.8	18.7	13.3	0.00131	0.5164	-0.9534
TDCA	Taurodeoxycholic acid	106	100	89	82.2	57.8	55.6	30.4	32.8	53.8	46.2	0.003629	0.503	-0.9914
TLCA	taurolithocholic acid	2.02	2.58	1.98	1.79	1.59	2.62	1.19	0.986	3.06	0.938	0.646314	0.8829	-0.1797
TUDCA	Tauroursodeoxycholic acid	26.4	24.8	105	101	73.6	87.4	76.4	38.8	88	44.4	0.968367	1.0127	0.0182
Tβ-MCA	Tauro-β-muricholic acid	22.2	21.4	46.4	48	38.2	19.1	18	14.1	23.6	21.2	0.046777	0.5448	-0.8762
UCA	Ursocholic acid	15	12.4	27.4	21.2	19.6	39.8	14.1	13.1	45	0.724	0.716145	1.1791	0.2377
α-MCA	α-muricholic acid	111	104	486	312	452	334	222	129	394	3.58	0.496466	0.739	-0.4364
β-MCA	β-muricholic acid	177	163	608	444	544	312	442	122	356	31.6	0.295316	0.6527	-0.6155
ω-MCA	ω-muricholic acid	113	87	348	238	372	244	304	41.6	246	19.7	0.484677	0.7386	-0.4371

Abbreviations:

P: P-value obtained from a two-tailed independent sample t-test. $P < 0.05$ indicates statistically significant differences between groups.

FC (Fold Change): The ratio of the average value in the treatment group to the control group. $FC > 1$ indicates upregulation; $FC < 1$ indicates downregulation.

Log_2FC: The base-2 logarithm of the fold change. $Log_2FC > 0$ indicates an increase; $Log_2FC < 0$ indicates a decrease. Log_2 transformation allows symmetrical interpretation of up- and down-regulation.

N/A: Data not available for the corresponding entry.

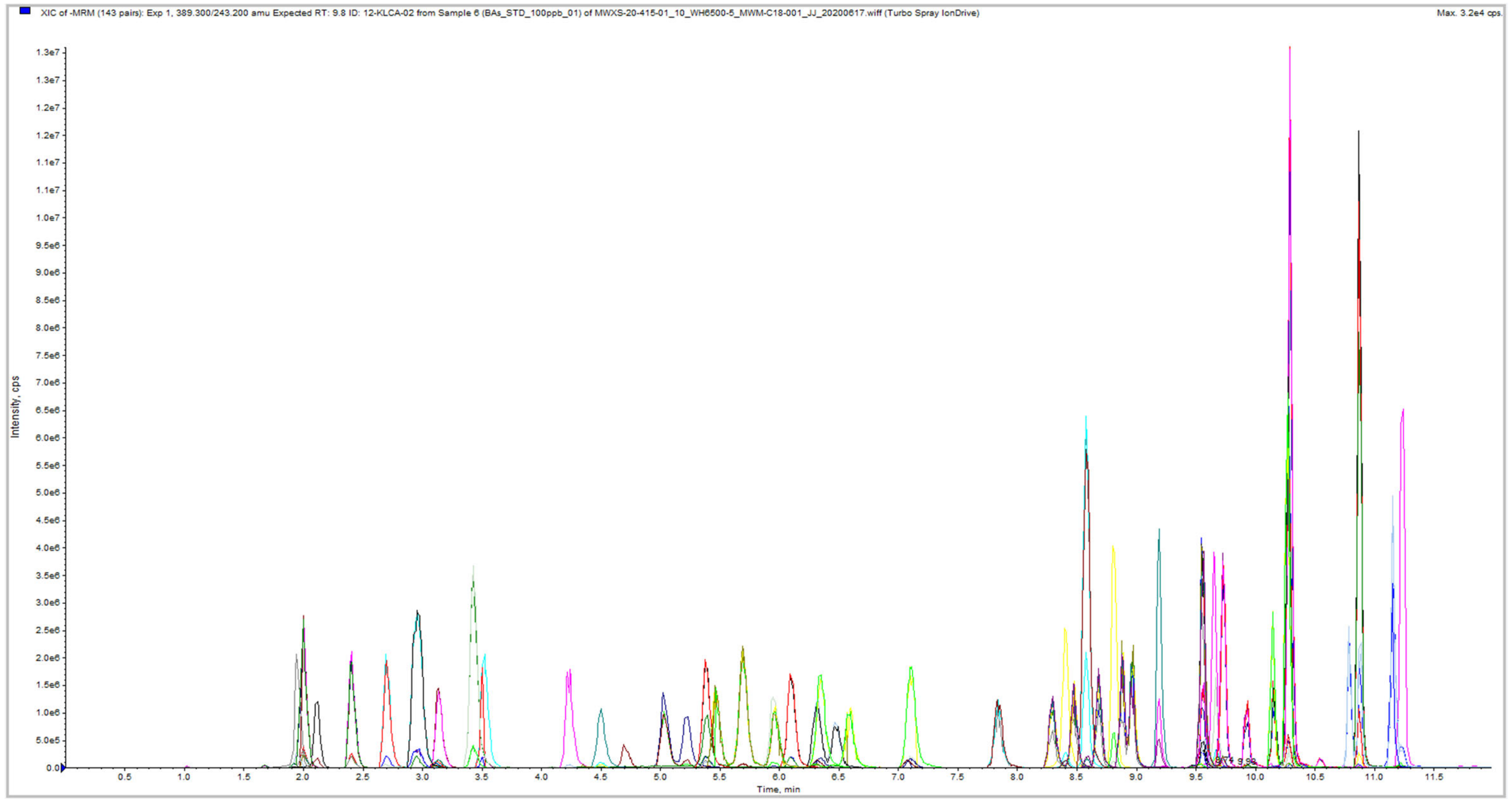

Figure S1. Multiple reaction monitoring (MRM) chromatogram of detected metabolites.

The multiple reaction monitoring (MRM) chromatogram provides a comprehensive visualization of metabolites detected within the samples. Each distinctively colored mass spectrometry peak corresponds to an individual metabolite, reflecting the method's sensitivity and specificity in profiling complex metabolic compositions.

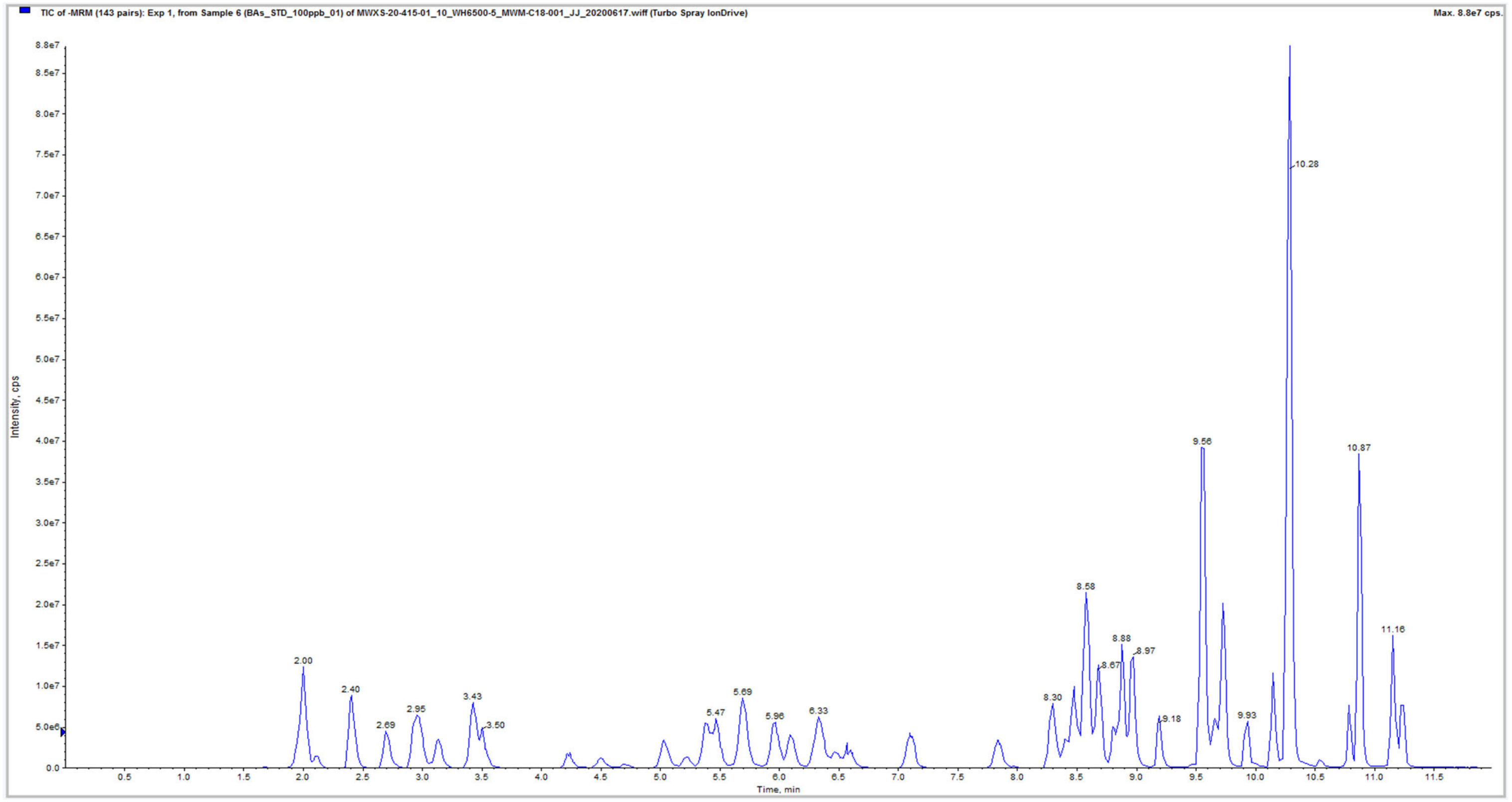

Figure S2. Total ion current (TIC) chromatogram of quality control (QC) samples

The TIC chromatogram demonstrates high reproducibility of bile acid detection. The overlapping TIC curves from multiple QC samples indicate consistent retention times and peak intensities, confirming instrumental stability and data reliability over repeated analyses.
